# Anti-Inflammatory Activities of Isogosferol, a Furanocoumarin Isolated from *Citrus junos* Seed Shells through Bioactivity-Guided Fractionation

**DOI:** 10.3390/molecules24224088

**Published:** 2019-11-12

**Authors:** Hwa Young Song, Ara Jo, Jihun Shin, Eui Hyeon Lim, Ye Eun Lee, Da Eun Jeong, Mina Lee

**Affiliations:** College of Pharmacy, Sunchon National University, 255 Jungangno, Suncheon-si 57922, Jeonnam, Korea; blueocean33@nate.com (H.Y.S.); coo123mm@naver.com (A.J.); wlgns0852@naver.com (J.S.); sksms147zld@naver.com (E.H.L.); qjsro1124@naver.com (Y.E.L.); dmsghktn333@naver.com (D.E.J.)

**Keywords:** *Citrus junos* seeds, coumarins, bioactivity-guided fractionation, inflammatory mediators, RAW 264.7 cells

## Abstract

*Citrus junos* Tanaka is a traditional medicine for treating coughs, dyspepsia, diabetes, asthma, neuralgia, and inflammatory disorders, and is distributed in Asia, especially in Korea, Japan, and China. This study aimed to use bioactivity-guided fractionation to find therapeutic phytochemicals from *C. junos* seeds, which can attenuate inflammatory responses. Nine coumarins (**1**–**9**) were isolated from the methanolic extract of *C. junos* seed shells and the inhibitory effects against inflammatory mediators were investigated using murine macrophages. Among the coumarins, compound **3**, isogosferol (ISO), more potently attenuated the production of nitric oxide (NO) in lipopolysaccharide (LPS)-induced RAW 264.7 cells. ISO also inhibited the expression of inducible nitric oxide (iNOS) and cyclooxygenase-2 (COX-2) in LPS-stimulated macrophages. Additionally, the phosphorylation of extracellular-regulated kinases (pERK)1/2 was reduced by ISO. We confirmed that ISO attenuated the release of interleukin-1 beta (IL-1β), which is a central mediator of the inflammatory response. These results demonstrate that ISO from *C. junos* seed shells may be a potent therapeutic candidate for the treatment of inflammatory diseases.

## 1. Introduction

Inflammation is an immune process that protects the human body against viral and bacterial infections [1]. Macrophages, mononuclear cells, and lymphocytes play critical roles in the inflammatory response [2], and the overexpression of nitric oxide (NO) and cytokines is associated with inflammatory diseases [3]. The expression of inducible nitric oxide (iNOS) is observed in response to bacterial lipopolysaccharide (LPS) and pro-inflammatory cytokines in diverse cell types, including macrophages, smooth muscle cells, hepatocytes, and astrocytes, and causes several diseases, such as sepsis, inflammation, and strokes [4,5]. Cyclooxygenase-2 (COX-2) is an enzyme that catalyzes the formation of prostaglandin E2 (PGE2), a precursor of inflammatory mediators, thus playing a pivotal role in mediating inflammatory responses [6]. Studies show that COX-2 inhibitors reduce cancer symptoms and inflammation [7]. LPS stimulates mitogen-activated protein kinases (MAPKs), such as ERK1/2 [8]. ERK1/2 plays important roles in the inflammatory response by regulating pro-inflammatory cytokines, including interleukin-1 beta (IL-1β) [9], which mediates the expression of iNOS in a variety of cell types [10]. In this study, we investigated the anti-inflammatory activity of coumarins isolated from *C. junos* via bioactivity-guided fractionation using LPS-activated murine macrophages. All of these factors can be considered key targets in the inhibition of inflammatory diseases.

When studying inflammation, it is important to consider oxidative stress, which is the production of free radicals and reactive oxygen species that are seen in a variety of conditions, including diabetes, aging, and degenerative nervous system diseases [11,12]. Excessive and continuous oxidative stress induces the expression of certain genes inside cells, leading to apoptosis as well as degenerative diseases causing chronic inflammation [13]. 

*C. junos*, a plant belonging to the family Rutaceae, has been used to treat fever and serious heart disease [14,15]. It is known to have anti-cancer, anti-inflammatory, anti-oxidant, anti-hyperglycemic, anti-bacterial, anti-viral, and anti-pyretic effects. It also has larvicidal and osteoclastogenic inhibitory effects [16]. The known chemicals isolated from *C. junos* include coumarins, limonoids, flavonoids, and triterpenoids [17]. The actual use of *C*. *junos* is generally limited to the flesh and peel, leaving unused seeds. Therefore, research on seed use is deemed necessary and this study aimed to elucidate the bioactivities of *C. junos* seeds and its phytochemicals. We evaluated the anti-inflammatory and anti-oxidant effects of *C. junos* seeds, oil extracted from them, and seed shells after removal of oil, and isolated various compounds from the most potent sub-fractions using bioactivity-guided fractionation. In addition, we determined the anti-inflammatory mechanism of coumarins isolated from the *C. junos* seeds without oil in LPS-induced macrophages, RAW 264.7 cells.

## 2. Results

### 2.1. Effects of C. junos Seed Extracts and Oil on NO Production and Cell Viability

**Figure 1:**
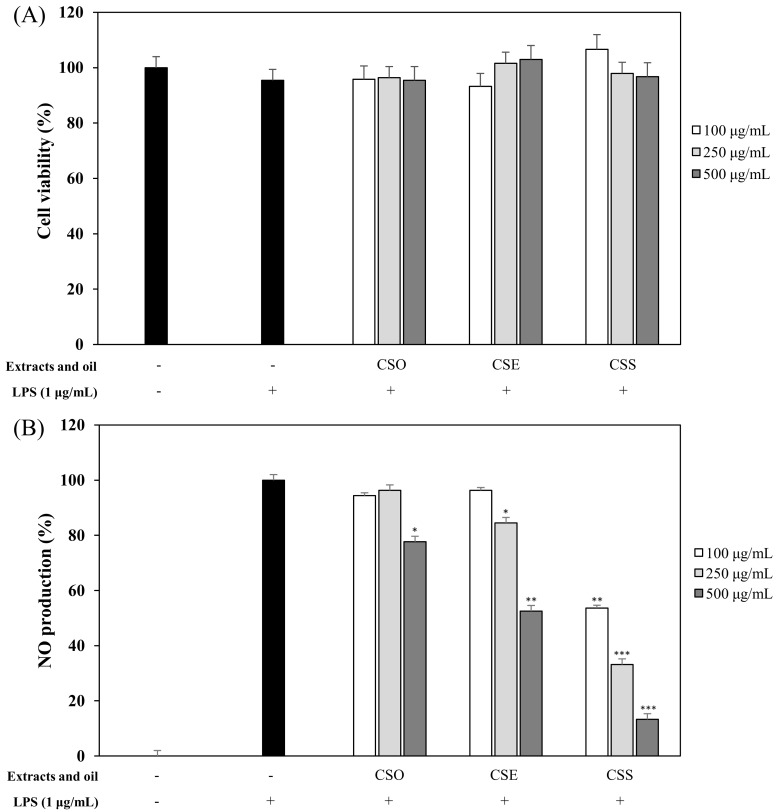
Effects of *Citrus junos* (*C. junos*) seed extracts and oil on cell viability (**A**) and nitric oxide (NO) production (**B**). RAW 264.7 cells were treated with extracts and oil (100, 250, 500 µg/mL). The samples are *C. junos* seeds extract (CSE), *C. junos* seeds oil (CSO), and *C. junos* seed shells extract without oil (CSS). Cell viability and the amount of nitrite in the culture medium were measured by 3-(4,5-dimethyl-2-thiazolyl)-2,5-diphenyl tetrazolium bromide (MTT) assay and Griess reagents, respectively. Nitrite concentrations of non-treated and lipopolysaccharide (LPS)-treated controls were 0.6 ± 0.1 μM and 16.5 ± 0.2 μM, respectively. The data are expressed as the mean ± SD (n = 3) of three individual experiments, and * *p* < 0.05, ** *p* < 0.01, and *** *p* < 0.001, compared with LPS-treated controls.

The anti-inflammatory activity of *C. junos* seeds was investigated. For sample preparation, pulverized *C. junos* seeds were extracted with 100% methanol and referred to as *C. junos* seed extracts (CSE). Also, *C. junos* seeds were known to contain oil and so the essential oil was extracted by supercritical fluid extraction (SFE) and this was called *C. junos* seeds oil (CSO). After removal of the oil, we repeated the extraction with 100% methanol and the acquired material was called *C. junos* seed shells extract without oil (CSS). The anti-inflammatory effects of CSE, CSO, and CSS were evaluated by studying the inhibition of NO production in LPS-induced RAW 264.7 cells. CSE, CSO, and CSS suppressed the NO production at 100, 250, and 500 μg/mL, in a concentration-dependent manner without inducing cytotoxicity (Figure 1). The CSS had the most potent inhibitory activity by reducing NO production by 60.5% at a concentration of 250 μg/mL (*p* < 0.001).

### 2.2. DPPH and ABTS Radical Scavenging Activities of CSE, CSO, and CSS

**Figure 2:**
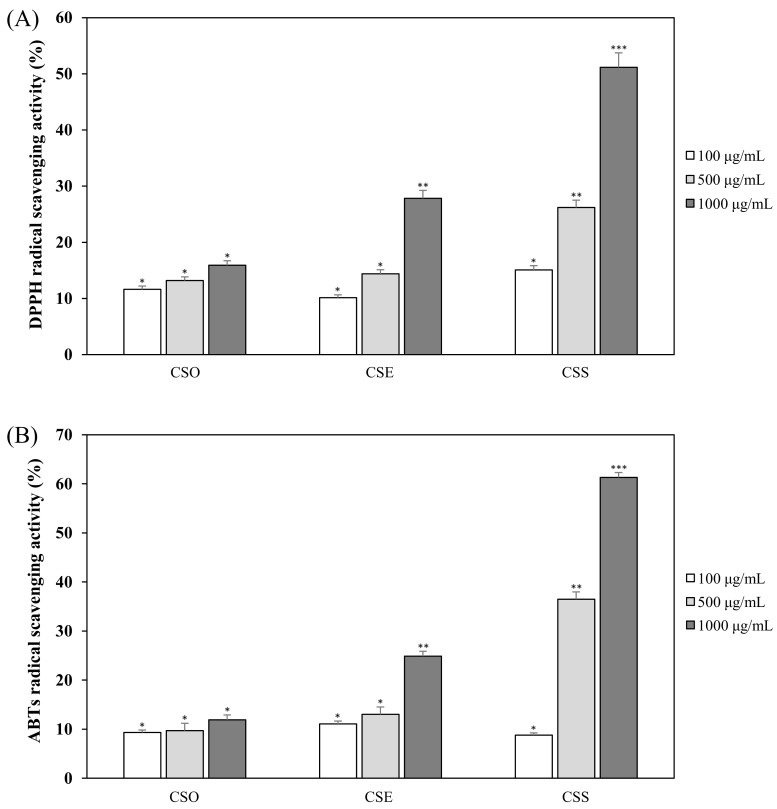
The 2,2-diphenyl-1-picrylhydrazyl (DPPH) radical scavenging activities (**A**) and 2,2′-azino-bis (ABTS) radical scavenging activities (**B**) of CSE, CSO, and CSS. The data are expressed as the mean ± SD (*n* = 3) of three individual experiments, and * *p* < 0.05, ** *p* < 0.01, and *** *p* < 0.001, compared with LPS-treated controls.

To identify the anti-oxidant activity of the *C. junos* seed extracts and oil, we evaluated the 2,2-diphenyl-1-picrylhydrazyl (DPPH) and 2,2′-azino-bis (ABTS) radical scavenging activities of CSE, CSO, and CSS at 100, 500, and 1000 μg/mL. With DPPH scavenging activity, the CSS and CSE showed concentration-dependent anti-oxidant effects at 100, 500, and 1000 μg/mL. At a concentration of 1000 μg/mL, the DPPH scavenging activities of CSS, CSE, and CSO were 75.7, 32.2, and 15.9%, respectively (Figure 2A). CSS had higher anti-oxidant activity than the others and CSO showed low effects. The ABTS radical scavenging activity was similar to that of the DPPH scavenging activity (Figure 2B). The ABTS scavenging activity of CSS, CSE, and CSO was 61.3, 24.9, and 11.9% at a concentration of 1000 μg/mL, respectively.

### 2.3. Bioactivity-Guided Isolation of Active Phytochemicals from CSS

**Figure 3:**
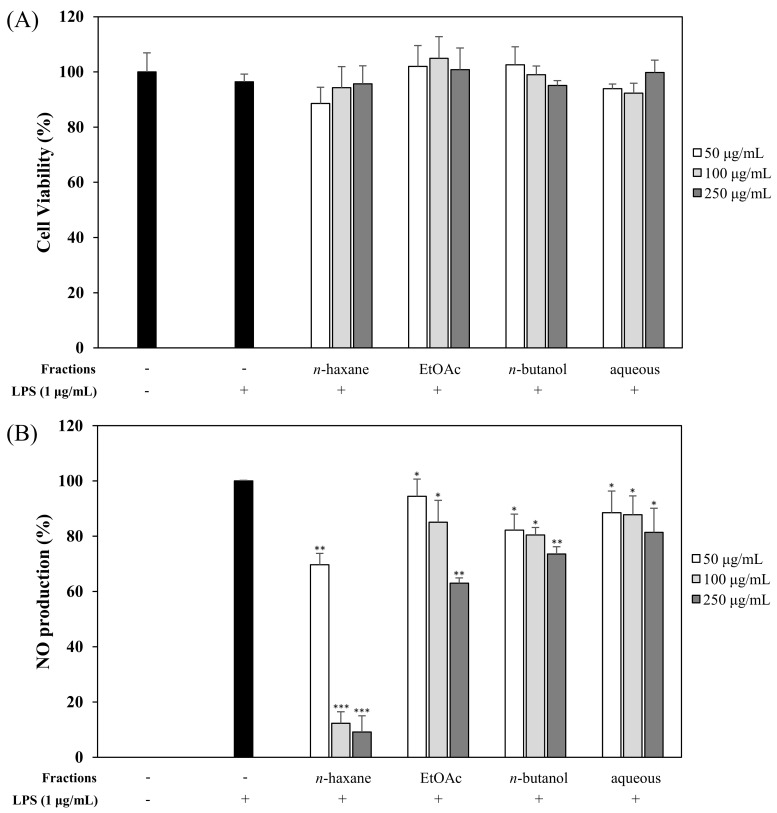
Effects of the CSS fractions on cell viability (**A**) and NO production (**B**). RAW 264.7 cells were treated with fractions (50, 100, and 250 µg/mL). Cell viability and the amount of nitrite in the culture medium were measured by MTT assay and Griess reagent, respectively. Nitrite concentrations of non-treated and LPS-treated controls were 0.6 ± 0.1 μM and 16.5 ± 0.2 μM, respectively. The data are expressed as the mean ± SD (*n* = 3) of three individual experiments, and * *p* < 0.05, ** *p* < 0.01, and *** *p* < 0.001, compared with LPS-treated controls.

**Figure 4:**
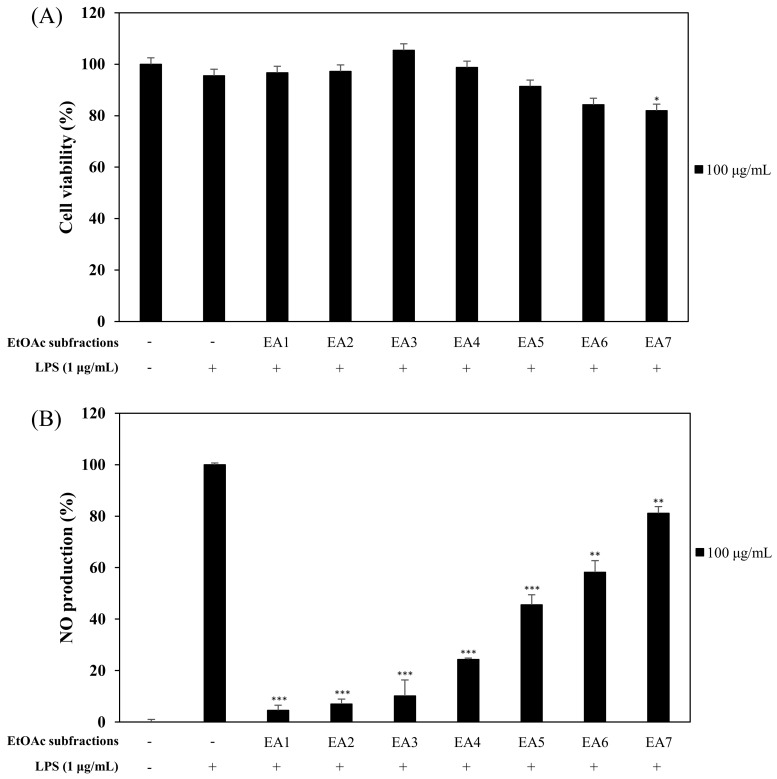
Effects of the ethyl acetate (EtOAc) subfractions (EA1-7) on cell viability (**A**) and NO production (**B**). RAW 264.7 cells were treated with EtOAc subfractions (100 µg/mL). Cell viability and the amount of nitrite in the culture medium were measured by MTT assay and Griess reagent, respectively. Nitrite concentrations of non-treated and LPS-treated controls were 0.6 ± 0.1 μM and 16.5 ± 0.2 μM, respectively. The data are expressed as the mean ± SD (*n* = 3) of three individual experiments, and * *p* < 0.05, ** *p* < 0.01, and *** *p* < 0.001, compared with LPS-treated controls.

**Figure 5:**
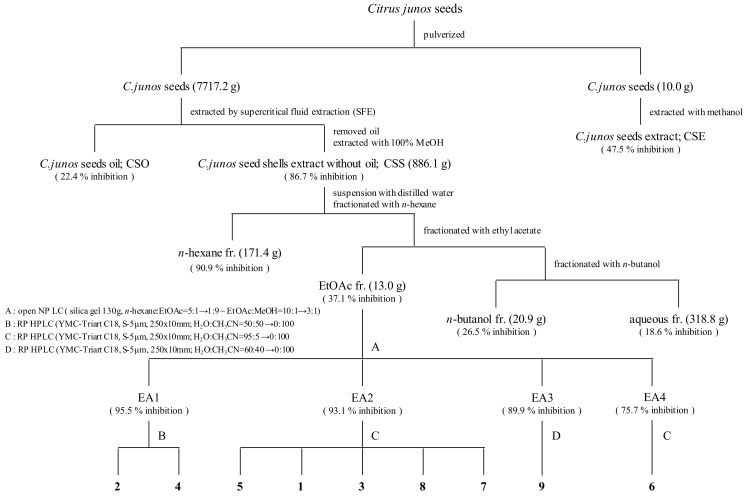
Procedure for isolation of compounds from *C. junos* seeds using bioactivity-guided fractionation. Nine compounds were isolated from *C. junos* seeds using bioactivity (inhibitory effects on NO production)-guided fractionation.

**Figure 6:**
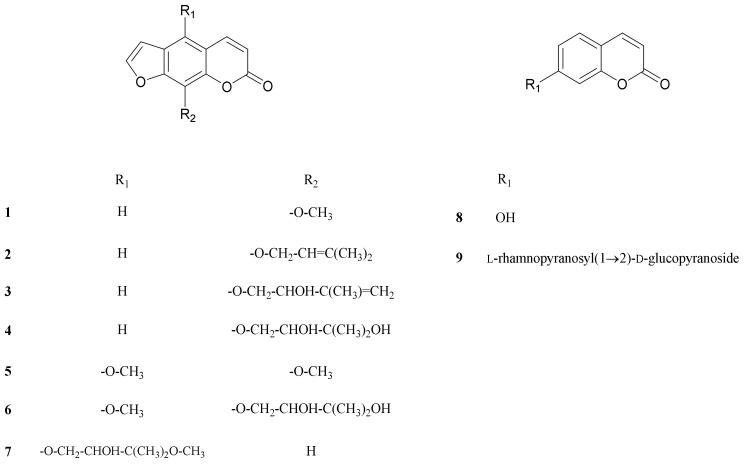
Structure of the nine compounds isolated from CSS.

We evaluated the effects of CSE, CSO, and CSS on anti-inflammation and anti-oxidant activities. Among them, the CSS significantly suppressed the NO production by 84.9% and the free radicals by 75.7% (Figure 1 and Figure 2). Based on bioactivity-guided fractionation, a large quantity of the CSS extract was partitioned with *n*-hexane, ethyl acetate (EtOAc), *n*-butanol, and aqueous residue, depending on the solvent polarity. All the fractions were measured to determine their effects on inhibition of NO production at 50, 100, and 250 μg/mL (Figure 3). When compared to all fractions, the *n*-hexane and EtOAc fractions down-regulated NO production in LPS-induced RAW 264.7 cells in a concentration-dependent manner. The EtOAc fraction was subjected to further extraction via bioactivity-guided isolation, while *n*-hexane was not since it contained a large amount of oil. The EtOAc fraction was separated through liquid chromatography and seven subfractions (EA1-7) were obtained. At 100 μg/mL, the EtOAc subfraction and EA1-7 all attenuated NO production in LPS-induced RAW 264.7 cells (Figure 4). In particular, EA1-4 showed 95.5, 93.1, 89.9, and 75.7% inhibition, respectively, without cytotoxicity, while EA5-7 had weak cytotoxicity. Therefore, we isolated the phytochemicals from EA1-4 via a reverse-phase (RP) multi-gradient high-performance liquid chromatography (HPLC) system. Bioactivity-guided fractionation was used to examine the inhibitory effects on NO production as shown in the schematic representation and this resulted in the isolation of nine compounds (Figure 5). These compounds were identified as (**1**) xanthotoxin, (**2**) imperatonin, (**3**) isogosferol, (**4**) heraclenol, (**5**) isopimpinellin, (**6**) byakangelicin, (**7**) heraclenol-3′ methyl ether, (**8**) umbelliferon, and (**9**) umbelliferon7-*O*-α-l-rhamnopyranosyl(1′,2)-β-d-glucopyranoside, by comparison of measured spectroscopic data with published papers (Figure 6) [18,19,20,21,22,23,24].

### 2.4. Effects of Nine Coumarins Isolated from CSS on NO Production and Cell Viability

**Figure 7:**
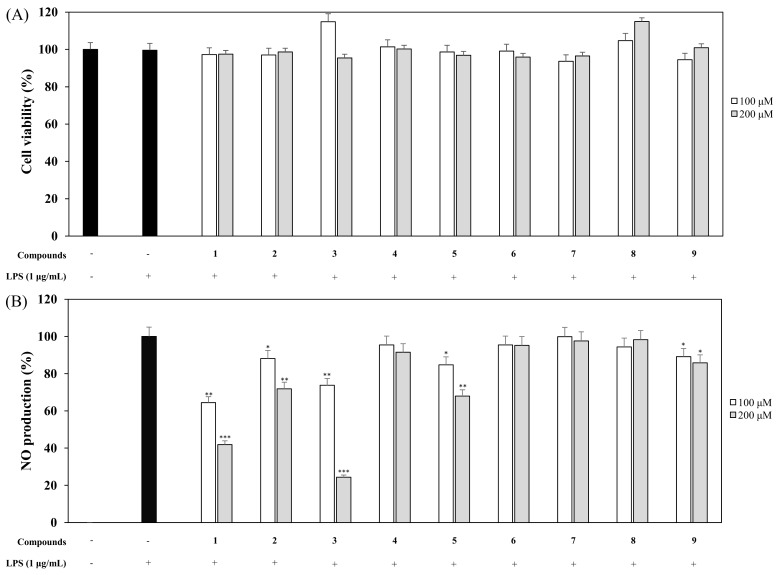
Effects of nine coumarins on cell viability (**A**) and NO production (**B**). RAW 264.7 cells were treated with nine coumarins (100 and 200 µM) for 1 h and stimulated with LPS (1 µg/mL) for 16 h. Cell viability and NO production were detected using an MTT assay and Griess reagent, respectively. Nitrite concentrations of non-treated and LPS-treated controls were 0.6 ± 0.1 μM and 16.5 ± 0.2 μM, respectively. The data are expressed as the mean ± SD (*n* = 3) of three individual experiments, and * *p* < 0.05, ** *p* < 0.01, and *** *p* < 0.001, compared with LPS-treated controls.

Macrophages were pretreated with nine coumarins at different concentrations (100 and 200 µM) for 1 h and stimulated with LPS (1 µg/mL) for 20 h. The supernatants (100 µL) were harvested and Griess reagent was used to measure the amount of nitrite ion present in the supernatant [25]. An 3-(4,5-dimethyl-2-thiazolyl)-2,5-diphenyl tetrazolium bromide (MTT) assay was used to measure cytotoxicity in macrophages that were pretreated with coumarins. The NO and cell viability results for the nine coumarins are shown in (Figure 7) and demonstrate that compound **3**, isogosferol (ISO), remarkably down-regulated the LPS-stimulated NO expression in comparison to the other compounds. The most potent compound, ISO, was with an IC_50_ of 148 μM followed by compound 1 with IC_50_ values of 166 μM and the IC_50_ values of other compounds were more than 200 μM. None of the coumarins affected cell viability, indicating that the inhibitory effects on NO levels did not contribute to cell viability. Since ISO had the most potent activity, it was used in all subsequent experiments.

### 2.5. Effects of ISO on LPS-Induced COX-2 and iNOS Expression

**Figure 8:**
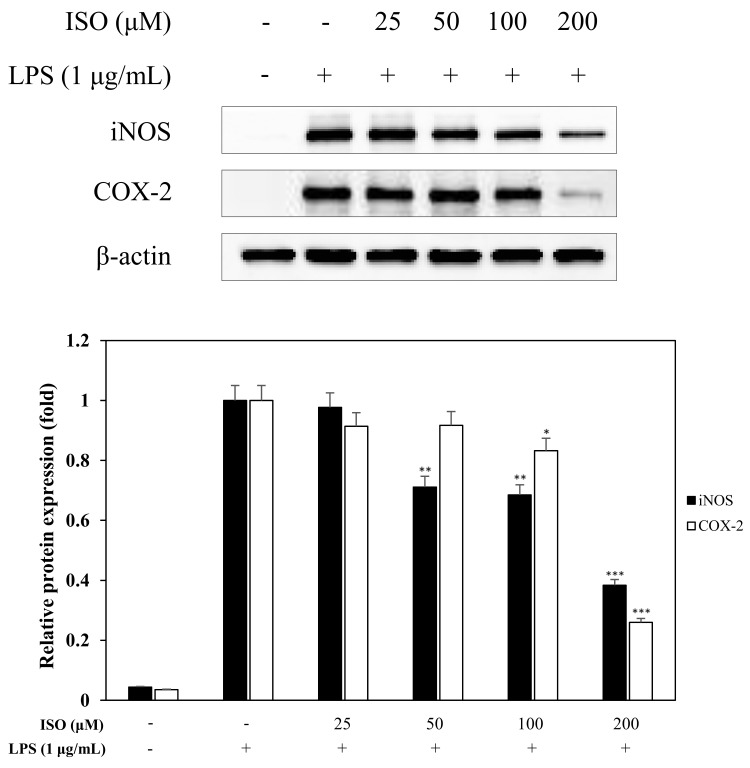
Effects of isogosferol (ISO) on iNOS and cyclooxygenase-2 (COX-2) expression. RAW 264.7 cells were incubated in the presence of ISO (25, 50, 100, and 200 µM) for 1 h and stimulated with LPS (1 µg/mL) for 16 h. The levels of iNOS, COX-2, and β-actin in the LPS-stimulated cells were analyzed by western blot analysis. Relative density was calculated as the ratio of each protein level compared to β-actin. The data are expressed as the mean ± SD (*n* = 3) of three individual experiments, and * *p* < 0.05, ** *p* < 0.01, and *** *p* < 0.001, compared with LPS-treated controls.

A western blot was performed to measure the effects of ISO on iNOS and COX-2, which cause several diseases including sepsis, inflammation, and strokes [26]. Upon LPS treatment for 16 h, iNOS activation was greatly increased in macrophages (RAW 264.7 cells). However, when macrophages were pretreated with ISO at concentrations of 25–200 μM before stimulation with LPS, there was a significant decrease in iNOS and COX-2 levels (Figure 8).

### 2.6. Effects of ISO on LPS-Induced pERK Production

**Figure 9:**
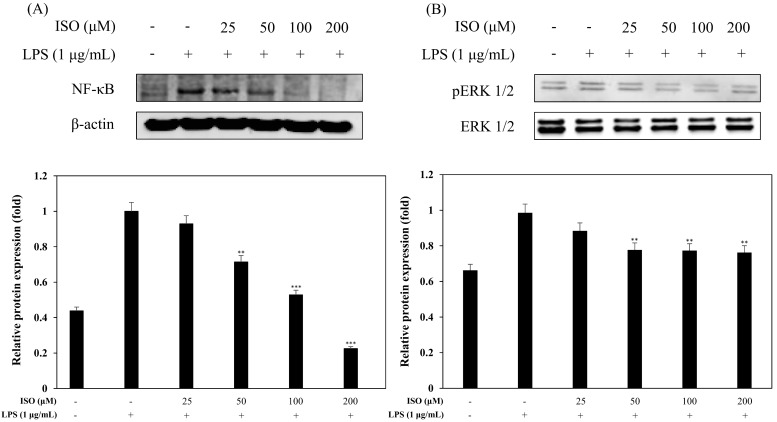
Effects of ISO on nuclear factor-kappa B (NF-κB) activation (**A**) and pERK1/2 (**B**) expression. RAW 264.7 cells were cultured in the presence of ISO (25, 50, 100, and 200 µM) for 1 h and stimulated with LPS (1 µg/mL) for 1 h. The levels of NF-κB p65 and pERK1/2 were detected by western blot analysis. The results are representative of three independent experiments. Relative density was calculated as the ratio of each protein compared to β-actin. The data are expressed as the mean ± SD (*n* = 3) of three individual experiments, and ** *p* < 0.01, and *** *p* < 0.001, compared with LPS-treated controls.

To confirm the effects of ISO on pERK1/2 production via western blot analysis, we pretreated RAW 264.7 cells with ISO at various concentrations (25, 50, 100, and 200 μM) for 1 h and then induced with LPS (1 μg/mL) for 1 h. Compared with the LPS-stimulated group, the LPS and ISO-treated group down-regulated the release of pERK1/2 in an ISO concentration-dependent manner from 25 μM to 200 μM (Figure 9A).

### 2.7. Effects of ISO on LPS-Induced NF-κB Activation

The nuclear factor-kappa B (NF-κB) transcription factor plays a role in regulating host inflammatory responses, cellular growth properties, and immune responses by increasing gene expression of *IL-1β, IL-2, IL-6*, *IL-8*, and other pro-inflammatory cytokines [27,28]. We wanted to determine if ISO could lead to reduced NF-κB protein levels [29], and the results confirmed reduced NF-κB production by western blot analysis (Figure 9B).

### 2.8. Inhibitory Effects of ISO on Cytokine Production

**Figure 10:**
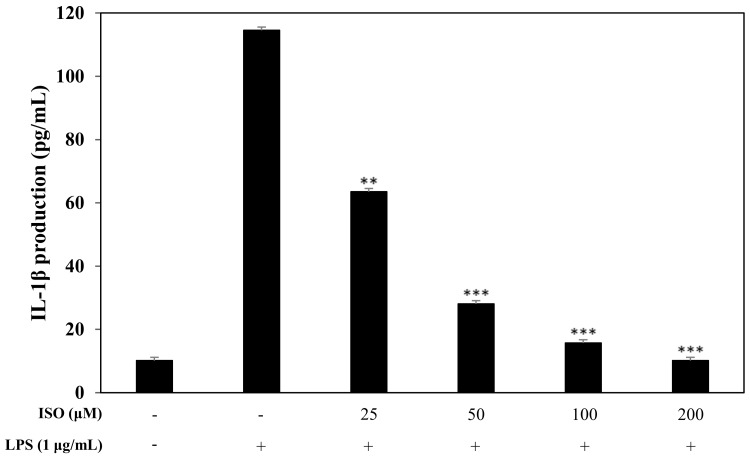
Effect of isogosferol on the release of the pro-inflammatory cytokine, IL-1β. RAW 264.7 cells were incubated with ISO (50, 100, and 200 µM) for 1 h and stimulated with LPS (1 µg/mL) for 16 h. Levels of IL-1β in the culture media supernatant were measured by ELLISA. The data are expressed as the mean ± SD (*n* = 3) of three individual experiments, and ** *p* < 0.01, and *** *p* < 0.001, compared with LPS-treated controls.

Pro-inflammatory cytokines, such as IL-1β, are pivotal markers of the inflammatory response. Since we knew that ISO influenced transcription factors, we also wanted to investigate the downstream impact on pro-inflammatory cytokines. Using an ELISA, we confirmed that ISO at 50, 100, and 200 μM greatly reduced IL-1β protein levels (Figure 10).

## 3. Discussion

The increase in the number of patients with inflammatory diseases is an important global health problem. Thus, there is a demand for developing protective and effective methods for addressing the problem. We explored potential therapeutic components from natural products. *C. junos*, also called ‘Yuja’ in Korean, is being cultivated on the southern coasts of Korea, producing quantities similar to fruits. However, the use of *C. junos* is commonly limited to the flesh and peel, while the seeds are usually discarded. The annual seeds waste exceeds 1800 tons in Korea. Therefore, discovering that the seeds are a high-value material through research could be both essential and meaningful. In this study, we investigated the applicability of *C. junos* seeds through the elucidation of its bioactivities.

We used *C. junos* seeds that were cultivated in Goheung, which is located on the southern coast of Korea and produces the largest amount of *C. junos* in Korea. The extract from *C. junos* seeds was studied to determine its bioactivity. The oil from *C. junos* seeds is used in cosmetic applications. For this study, the oil was obtained by SFE and also investigated for its bioactive effects. In addition, we found that seed shells were also not being used and contain some oil. Therefore, *C. junos* seed shells without oil were also investigated to determine if their value may be higher than expected.

At first, the inhibitory effects of CSE, CSO, and CSS on NO production were measured in LPS-induced macrophages, and we confirmed that CSS suppressed the production of NO much better than the other extracts (Figure 1). Next, we investigated the DPPH and ABTS radical scavenging activities of CSE, CSO, and CSS because excessive and continuous oxidative stress is known to be related to inflammatory responses (Figure 2). The results showed that CSS had higher anti-inflammatory and anti-oxidant activities than CSE or CSO. Inflammatory activity-guided fractionation, by assessing the inhibitory effects on NO production, was used to partition the CSS using *n*-hexane, ethyl acetate, *n*-butanol, H_2_O, followed by fractionation again with EtOAc to obtain bioactive subfractions (Figure 3, Figure 4 and Figure 5). To discover anti-inflammatory phytochemicals, we tested whether coumarins (1–9) isolated from these subfractions could inhibit the production of NO in LPS-induced RAW 264.7 cells (Figure 6 and Figure 7). Of the nine coumarins, compound 3, ISO, attenuated NO production by 75.7%. We performed subsequent experiments with the ISO.

Since iNOS and COX-2 regulate NO and PGE2 production during the inflammatory response [30], causing the pathogenesis of a variety of inflammatory diseases [31], we examined the inhibitory effects of ISO on iNOS and COX-2 by western blot (Figure 8) and saw less iNOS and COX-2. Activation of iNOS and COX-2 is accompanied by the release of pro-inflammatory cytokines, such as IL-1β in LPS-induced macrophages [32]. Almost all infectious agents lead to the release of IL-1β from many cell types, including macrophages [33]. Modulating the expression of pro-inflammatory cytokines is important for affecting the inflammatory response [34,35,36]. Hence, to assess the inhibitory effects of ISO on the production of IL-1β, we examined IL-1β levels following LPS stimulation of RAW 264.7 cells (Figure 10). Our results indicated that ISO inhibited the activation of IL-1β. Extracellular signal-regulated protein kinases 1 and 2 (ERK1/2), MAPKs, are molecules which play important roles in cell signaling pathways involved in inflammation, apoptosis, and cell death [37]. Therefore, we investigated the influence of ISO on the phosphorylation of ERK1/2 (Figure 9). This result showed that ISO suppressed the phosphorylation of ERK1/2, revealing anti-inflammatory effects.

Based on current literature, our results showed that extracts and oil derived from *C. junos* seed shells have anti-inflammatory and anti-oxidant effects. ISO, a phytochemical isolated from *C. junos* seed shells using bioactivity-guided fractionation, suppressed NO levels and down-regulated the activation of the pro-inflammatory cytokine IL-1β and other inflammatory mediators, including phospho-nuclear factor-kappa B (pNF-κB), pERK1/2, iNOS, and COX-2, using an LPS-stimulated macrophage cell line, RAW 264.7 cells. This study suggests that ISO, a constituent of *C. junos* seed shells, could be useful as a candidate anti-inflammatory agent. Through this study, we discovered a new potential of use, rather than simply discarding them for material that is thought to be valuable.

## 4. Materials and Methods

### 4.1. Plant Materials

*C. junos* seeds were purchased from Shinkiwon, a social enterprise (Goheung, Korea), in 2017. *C. junos* seeds were pulverized and seed shells were obtained. The essential oil was extracted from them by SFE, which was performed under pressure at 280 bar at 40 °C. Using 100% methanol, we performed extractions on the seeds and seed shells after removal of oil, and then these were called *C. junos* seeds extract (CSE) and *C. junos* seed shells extract without oil (CSS), respectively. The CSE, CSO, and CSS were provided by the Jeonnam Nanobio Research Center.

### 4.2. Extraction and Isolation

Oil was removed from the pulverized *C. junos* Tanaka seeds (7717.2 g) by SFE and extracted three times with 100% methanol for 2 h, using sonication. The extracted solution was filtered and dried to produce the methanol extract (886.1 g). The extract was suspended in distilled H_2_O and polar-sequentially partitioned with *n*-hexane, EtOAc, and *n*-butanol to obtain 171.4 g, 13.0 g, 20.9 g, and 318.8 g of residue, respectively. The EtOAc fraction showed a significant inhibitory effect on NO production in RAW 264.7 cells and was therefore used for further isolation work. The EtOAc fraction was separated by open liquid chromatography (LC) using a gradient solvent system in normal phase (NP) liquid column chromatography with 13 g of silica gel and an *n*-hexane:EtOAc ratio of 5:1 into 100% methanol to obtain seven subfractions (EA1-7). The EA1 was separated using an RP-HPLC system, with a YMC Triart C_18_ 250 × 10 mm column, and a H_2_O:CH_3_CN ratio of 1:1 into 100% acetonitrile, at a rate of 2 mL/min. Compound 2 (1.6 mg, *t*_R_ 45.2 min, H_2_O:acetonitrile = 24.7:75.3) and compound 4 (2.1 mg, *t*_R_ 15.2 min, H_2_O:acetonitrile = 55:45) were obtained. The EA2 was subjected to RP-HPLC using the YMC Triart C_18_ column and a H_2_O:acetonitrile ratio of 95:5 into 100% acetonitrile at a rate of 2 mL/min to obtain five compounds, including compound 5 (18 mg, *t*_R_ 36.4 min, H_2_O:acetonitrile = 17.2:82.8), 1 (2 mg, *t*_R_ 34.7 min, H_2_O:acetonitrile = 20.6:79.4), 3 (0.5 mg, *t*_R_ 33.9 min, H_2_O:acetonitrile = 22.2:77.8), 8 (2.2 mg, *t*_R_ 27.2 min, H_2_O:acetonitrile = 35.6:64.4), and 7 (1.3 mg, *t*_R_ 32.9 min, H2O:acetonitrile = 24.2:75.8), in order of polarity. Compound 9 (5 mg, *t*_R_ 9.2 min, H2O:acetonitrile = 60:40) from EA3 and 6 (3.5 mg, *t*_R_ 32.4 min, H_2_O:acetonitrile = 35.2:64.8) from EA4 were isolated through HPLC using the YMC Triart C_18_ column at respective H_2_O:acetonitrile ratios of 60:40 and 92:8 into 100% acetonitrile at a rate of 2 mL/min. To detect these chromatograms, it was used as a wavelength of 210 nm, 254 nm, and 365 nm.

### 4.3. DPPH Assay

The radical scavenging effects of DPPH (2,2-diphenyl-1-picrylhydrazyl, Sigma-Aldrich, St. Louis, MO, USA) were evaluated by a modified Blois method [38]. DPPH reagent (100 μL of 0.6 mM) was added to 100 μL samples of CSS, CSE, or CSO in 96-well plates, mixed for 25 s, and incubated for 30 min while covering to protect from light. Absorbances at 517 nm were measured using a microplate reader (BioTek Instruments, Winooski, VT, USA).

### 4.4. ABTS Assay

The ABTS (2,2-Azino-bis(3-ethylbenzothiazoline-6-sulfonic acid diammonium salt, Sigma-Aldrich, Co.) radical scavenging activity was evaluated by a modified Blois method [39]. The ABTS solution (100 μL of 7.4 mM) was added to 100 μL samples (CSS, CSE, or CSO) in a 96-well plate, mixed for 25 s, and incubated for 6 min while protecting from light. The absorbance was measured at 734 nm using a microplate reader (BioTek Instruments, Winooski, VT, USA).

### 4.5. Cell Culture

RAW 264.7 cells, a macrophage cell line, were purchased from the Korean Cell Line Bank (Seoul, Korea). The macrophages were maintained as a monolayer in Dulbecco’s modified Eagle’s medium supplemented with 10% fetal bovine serum (FBS), 100 IU/ml penicillin, and 100 µg/mL streptomycin solution (Hyclone, Logan, UT, USA) in a humidified environment with 5% CO_2_ at 37 °C.

### 4.6. Cell Viability Assay

RAW 264.7 cells were seeded at 1 × 10^5^ cells/well in 96-well plates and incubated for 24 h. Different concentrations of samples (CSE, CSO, CSS, fractions, or coumarins) were added to the cells and their cytotoxic effects were measured using an MTT (3-[4,5-dimethyl-2-thiazolyl]-2,5-diphenyl tetrazolium bromide) (Sigma-Aldrich, Saint Louis, MO, USA) assay. After 4 h, the supernatant was aspirated and 100 μL DMSO was added. The absorbance of dissolved formazan crystals was measured at 570 nm using a microplate reader (BioTek Instruments, Winooski, VT, USA).

### 4.7. Measurement of NO Production

Macrophages were plated at 1 × 10^5^ cells/well in 96-well plates and were treated with different concentrations of samples (CSE, CSO, CSS, fractions, or coumarins) for 1 h and then stimulated with LPS (1 μg/mL). After 20 h, 100 μL of supernatant from the macrophages was harvested and added to equal volumes of Griess reagent A (0.1% N-(1-naphthyl) ethylenediamine dihydrochloride in DW) and Griess reagent B (1% sulfanilamide in 5% phosphoric acid). The reaction was incubated in the dark at room temperature for 15 min and the absorbance was measured at 550 nm using a microplate reader (BioTek Instruments, Winooski, VT, USA). Serum-free culture medium was used as the blank in all experiments.

### 4.8. Western Blot Analysis

RAW 264.7 cells (1 × 10^6^ cells/well) were plated in 6-well plates. After 24 h, the cells were treated with various concentrations of ISO for 2 h and then stimulated with LPS (1 μg/mL). The cells were washed with cold phosphate-buffered saline (PBS) and the whole cell lysates were suspended in a protein extraction solution (proprep, iNtRON Biotechnology, Korea). Equal amounts of protein were separated by 10% sodium dodecyl sulfate (SDS)-PAGE, transferred to nitrocellulose membranes, and blocked with 5% skim milk in plain buffer (20 mM Tris, pH 7.4; and 136 mM NaCl) for 1 h. The membranes were incubated overnight at 4 °C in primary antibody solutions to measure iNOS, COX-2, NF-κB, and pERK1/2 proteins. The membranes were then incubated in horseradish peroxidase (HRP)-conjugated secondary antibody solutions for 2 h at room temperature while shaking. After washing, the western blot detection reagent (ECL solution) was added and bands were visible. The bands were captured and measured using a bio-imaging system (Microchemi 4.2 Chemilumineszenz-System, Neve Yamin, Israel).

### 4.9. Measurement of Cytokine Production

Levels of IL-1β in murine macrophage cells were determined by ELISA kits (BD Biosciences, San Diego, CA, USA) following the manufacturer’s instructions.

### 4.10. Statistical Analysis

Our data are expressed as means ± standard deviation (SD) of at least three independent experiments. One-way ANOVA was used for comparisons of multiple group means and statistical significance was considered at *p* < 0.05.

^1^H and ^13^C NMR spectra of nine compounds are available in Supplementary Materials.

## Supplementary Materials

The following are available online.^1^H and ^13^C NMR spectra of nine compounds.

## References

[1] Ryu H.W., Lee S.U., Lee S., Song H., Son T.H., Kim Y., Yuk H.J., Ro H., Lee C., Hong S. (2017). 3-Methoxy-Catalposide Inhibits Inflammatory Effects in Lipopolysaccharide-Stimulated RAW264. 7 Macrophages. Cytokine.

[2] Kim E., Kim S., Ye B., Kim J., Ko S., Lee W.W., Kim K., Choi I., Jung W., Heo S. (2018). Anti-Inflammatory Effect of Apo-9′-Fucoxanthinone Via Inhibition of MAPKs and NF-kB Signaling Pathway in LPS-Stimulated RAW 264.7 Macrophages and Zebrafish Model. Int. Immunopharmacol..

[3] Spranger J., Kroke A., Mohlig M., Hoffmann K., Bergmann M.M., Ristow M., Boeing H., Pfeiffer A.F. (2003). Inflammatory Cytokines and the Risk to Develop Type 2 Diabetes: Results of the Prospective Population-Based European Prospective Investigation into Cancer and Nutrition (EPIC)-Potsdam Study. Diabetes.

[4] Lee S.H., Lee S.Y., Son D.J., Lee H., Yoo H.S., Song S., Oh K.W., Han D.C., Kwon B.M., Hong J.T. (2005). Inhibitory Effect of 2′-Hydroxycinnamaldehyde on Nitric Oxide Production through Inhibition of NF-κB Activation in RAW 264.7 Cells. Biochem. Pharmacol..

[5] Nathan C. (1992). Nitric Oxide as a Secretory Product of Mammalian Cells. FASEB J..

[6] Kim J., Park S.J., Yun K., Cho Y., Park H., Lee K. (2008). Isoliquiritigenin Isolated from the Roots of Glycyrrhiza Uralensis Inhibits LPS-Induced iNOS and COX-2 Expression Via the Attenuation of NF-κB in RAW 264.7 Macrophages. Eur. J. Pharmacol..

[7] Levy G.N. (1997). Prostaglandin H Synthases, Nonsteroidal Anti-Inflammatory Drugs, and Colon Cancer. FASEB J..

[8] Jin M.H., Suh S.J., Yang J.H., Lu Y., Kim S.J., Kwon S.Y., Jo T.H., Kim J.W., Park Y.I., Ahn G.W. (2010). Anti-Inflammatory Activity of Bark of Dioscorea Batatas DECNE through the Inhibition of iNOS and COX-2 Expressions in RAW264. 7 Cells Via NF-κB and ERK1/2 Inactivation. Food Chem. Toxicol..

[9] Zhu Z., Gu Y., Zhao Y., Song Y., Li J., Tu P. (2016). GYF-17, a Chloride Substituted 2-(2-Phenethyl)-Chromone, Suppresses LPS-Induced Inflammatory Mediator Production in RAW264. 7 Cells by Inhibiting STAT1/3 and ERK1/2 Signaling Pathways. Int. Immunopharmacol..

[10] Finder J.D., Petrus J.L., Hamilton A., Villavicencio R.T., Pitt B.R., Sebti S.M. (2001). Signal Transduction Pathways of IL-1β-Mediated iNOS in Pulmonary Vascular Smooth Muscle Cells. Am. J. Physiol. Lung Cell. Mol. Physiol..

[11] Reuter S., Gupta S.C., Chaturvedi M.M., Aggarwal B.B. (2010). Oxidative Stress, Inflammation, and Cancer: How are they Linked?. Free Radic. Biol. Med..

[12] Maritim A., Sanders A., Watkins Iii J. (2003). Diabetes, Oxidative Stress, and Antioxidants: A Review. J. Biochem. Mol. Toxicol..

[13] Kang H. (2012). Antioxidant and Anti-Inflammatory Effect of Extracts from Flammulina Velutipes (Curtis) Singer. J. Korean Soc. Food Sci. Nutr..

[14] Kim K., Cho H., Jung H., Lee H.J., Hwang K.T. (2017). Anti-Proliferative Effect of Methanolic Extracts from Citrus Junos Seeds and Seed Oils on HT-29 Human Colon Cancer Cells and Identification of their Major Bioactive Compounds. Korean J. Food Sci. Technol..

[15] Lee S.H., Lee M.S. (2017). The Study of Physiological and Antimicrobial Activities on the Citrus Junos Extracts with its Textures and Skin. J. Korea Acad. Ind. Coop. Soc..

[16] Gualdani R., Cavalluzzi M.M., Lentini G., Habtemariam S. (2016). The Chemistry and Pharmacology of Citrus Limonoids. Molecules.

[17] Shinoda N., Shiga M., Nishimura K. (1970). Constituents of Yuzu (Citrus Junos) Oil. Agric. Biol. Chem..

[18] Fujioka T., Furumi K., Fujii H., Okabe H., Mihashi K., Nakano Y., Matsunaga H., Katano M., Mori M. (1999). Antiproliferative Constituents from Umbelliferae Plants. V. A New Furanocoumarin and Falcarindiol Furanocoumarin Ethers from the Root of Angelica Japonica. Chem. Pharm. Bull..

[19] Wang G., Zhou Z., Cheng C., Yao J., Yang Z. (2008). Osthol and Isopimpinellin from Fructus Cnidii for the Control of Dactylogyrus Intermedius in Carassius Auratus. Vet. Parasitol..

[20] Liu R., Feng L., Sun A., Kong L. (2004). Preparative Isolation and Purification of Coumarins from Cnidium Monnieri (L.) Cusson by High-Speed Counter-Current Chromatography. J. Chromatogr. A.

[21] Luz R.F., Vieira I.J., Braz-Filho R., Moreira V.F. (2015). 13C-NMR Data from Coumarins from Moraceae Family. Am. J. Anal. Chem..

[22] Kim B., Jung E., Youn H., Hwang K., Han M., Yu Y., Nam K. (2017). Inhibitory Effects of Coumarins from the Fruit of Citrus Aurantium Against iNOS Activity. Yakhak Hoeji.

[23] Setzer W.N., Vogler B., Bates R.B., Schmidt J.M., Dicus C.W., Nakkiew P., Haber W.A. (2003). HPLC-NMR/HPLC-MS Analysis of the Bark Extract of Stauranthus Perforatus. Phytochem. Anal. Int. J. Plant. Chem. Biochem. Tech..

[24] Nam V.D., Teruhisa F., Hirofumi T., Hiroshi K., Khoi N.M., Dung L.V., Ha D.T., Hiroshi H. (2016). Chemical Composition of Clausena Lansium (Lour.) Skeels Leaves and Antifungal Activity. Nat. Prod. Sci..

[25] Zhang Y., Yi P., Chen Y., Mei Z., Hu X., Yang G. (2014). Lycojaponicuminol A–F: Cytotoxic Serratene Triterpenoids from Lycopodium Japonicum. Fitoterapia.

[26] Chen Y., Shen S., Lee W., Hou W., Yang L., Lee T.J. (2001). Inhibition of Nitric Oxide Synthase Inhibitors and Lipopolysaccharide Induced Inducible NOS and cyclooxygenase-2 Gene Expressions by Rutin, Quercetin, and Quercetin Pentaacetate in RAW 264.7 Macrophages. J. Cell. Biochem..

[27] Yamamoto Y., Gaynor R.B. (2001). Therapeutic Potential of Inhibition of the NF-kappaB Pathway in the Treatment of Inflammation and Cancer. J. Clin. Investig..

[28] Haefner B. (2002). NF-κB: Arresting a Major Culprit in Cancer. Drug Discov. Today.

[29] Baeuerle P.A. (1991). The Inducible Transcription Activator NF-κB: Regulation by Distinct Protein Subunits. Biochim. Biophys. Acta (BBA) Rev. Cancer.

[30] Han S., Lee J.H., Kim C., Nam D., Chung W., Lee S., Ahn K.S., Cho S.K., Cho M., Ahn K.S. (2013). Capillarisin Inhibits iNOS, COX-2 Expression, and Proinflammatory Cytokines in LPS-Induced RAW 264.7 Macrophages Via the Suppression of ERK, JNK, and NF-κB Activation. Immunopharmacol. Immunotoxicol..

[31] Rockey D.C., Chung J.J., McKee C.M., Noble P.W. (1998). Stimulation of Inducible Nitric Oxide Synthase in Rat Liver by Hyaluronan Fragments. Hepatology.

[32] Kim H.K., Cheon B.S., Kim Y.H., Kim S.Y., Kim H.P. (1999). Effects of Naturally Occurring Flavonoids on Nitric Oxide Production in the Macrophage Cell Line RAW 264.7 and their structure–activity Relationships. Biochem. Pharmacol..

[33] Dinarello C.A. (1996). Biologic Basis for Interleukin-1 in Disease. Blood.

[34] Blonska M., Czuba Z., Krol W. (2003). Effect of Flavone Derivatives on Interleukin-1β (IL-1β) mRNA Expression and IL-1β Protein Synthesis in Stimulated RAW 264.7 Macrophages. Scand. J. Immunol..

[35] Wu C., Chen T., Chen T., Ho W., Chiu W., Chen R. (2003). Nitric Oxide Modulates Pro-and Anti-Inflammatory Cytokines in Lipopolysaccharide-Activated Macrophages. J. Trauma.

[36] Wischmeyer P.E., Kahana M., Wolfson R., Ren H., Musch M.M., Chang E.B. (2001). Glutamine Reduces Cytokine Release, Organ Damage, and Mortality in a Rat Model of Endotoxemia. Shock.

[37] Qi M., Elion E.A. (2005). MAP Kinase Pathways. J. Cell Sci..

[38] Blois M.S. (1958). Antioxidant Determinations by the use of a Stable Free Radical. Nature.

[39] Proestos C., Lytoudi K., Mavromelanidou O., Zoumpoulakis P., Sinanoglou V. (2013). Antioxidant Capacity of Selected Plant Extracts and their Essential Oils. Antioxidants.

