# Supplementary material for: Anti-Inflammatory Activities of Isogosferol, a Furanocoumarin Isolated from Citrus junos Seed Shells through Bioactivity-Guided Fractionation
Source: Molecules. 2019 Nov 12;24(22):4088. doi: 10.3390/molecules24224088 (PMC6891542; doi:10.3390/molecules24224088)
Supplement: Supplementary file 1 [file molecules-24-04088-s001.pdf]

Supplementary data

Isogosferol, a furanocoumarin isolated from *Citrus junos* seed shells using bioactivity-guided fractionation, inhibit the inflammatory mediators via ERK signaling

Hwa Young Song[1], Ara Jo[1], Jihun Shin, Eui Hyeon Lim, Ye Eun Lee, Da Eun Jeong and Mina Lee[*]

College of Pharmacy, Sunchon National University, 255 Jungangno, Suncheon-si 57922, Jeonnam, Republic of Korea

* Correspondence: Tel. 82-61-750-3764 Fax. 82-61-750-3708 E-mail: minalee@sunchon.ac.kr

Supporting Information Available

S1. ^{1}H-NMR (400 MHz, DMSO-d_6) data of coumarins

S2. ^{13}C-NMR (100 MHz, DMSO-d_6) data of coumarins

S1. ^{1}H-NMR (400 MHz, DMSO-d_6) data of coumarins

Compound **1**: δ 8.13 (1H, d, *J* = 10.0 Hz, H-4), 8.12 (1H, d, *J* = 2.4 Hz, H-1'), 7.67 (1H, s, H-5), 7.1 (1H, d, *J* = 1.6 Hz, H-2'), 6.43 (1H, d, *J* = 9.6 Hz, H-3), 4.2 (3H, s, OCH_3)

Compound **2**: δ 8.13 (1H, d, *J* = 9.6 Hz, H-4), 8.11 (1H, d, *J* = 2.4 Hz, H-1'), 7.68 (1H, s, H-5), 7.08(1H, d, *J* = 2.4 Hz, H-2 '), 6.42 (1H, d, *J* = 9.6 Hz, H-3), 5.50 (1H, t, *J* = 7.2 Hz, C-2''), 4.90 (2H, d, *J* = 7.2 Hz, C-1''), 1.68 (3H, s, H-4''), 1.63 (3H, s, H-5'')

Compound **3**: δ 8.14 (1H, d, *J* = 9.6 Hz, H-4), 8.12 (1H, d, *J* = 2.4 Hz, H-1'), 7.67 (1H, s, H-5), 7.09 (1H, d, *J* = 2.4 Hz, H-2'), 6.43 (1H, d, *J* = 9.6 Hz, H-3), 5.05 (1H, d, *J* = 1.2 Hz, H-4''α), 4.87 (1H, d, *J* = 2.0 Hz, H4''β), 4.37 (1H, m, H-2''), 4.32 (2H, m, H-1''), 1.75 (3H, s, H-5'')

Compound **4**: δ 8.13 (1H, d, *J* = 10.0 Hz, H-4), 8.12 (1H, d, *J* = 2.4 Hz, H-1'), 7.08 (1H, d, *J* = 2.4 Hz, H-2'), 6.42 (1H, d, *J* = 9.6 Hz, H-3), 4.56 (1H, dd, *J* = 10.0, 2.4 Hz, H-1''α), 4.33 (1H, dd, *J* = 10.2, 8.2 Hz, H-1''β), 3.78 (1H, m, H-2''), 1.14 (3H, s, H-4''), 1.06 (3H, s, H-5'')

Compound **5**: δ 8.17 (1H, d, *J* = 10.0 Hz, H-4), 8.08 (1H, d, *J* = 2.0 Hz, H-1'), 7.38 (1H, d, *J* = 2.4 Hz, H-2'), 6.74 (1H, d, *J* = 9.6 Hz, H-3), 4.16 (3H, s, OCH_3), 4.02 (3H, s, OCH_3)

Compound **6**: δ 8.18 (1H, d, *J* = 9.6 Hz, H-4), 8.08 (1H, d, *J* = 2.8 Hz, H-1'), 7.37 (1H, d, *J* = 2.4 Hz, H-2'), 6.33 (1H, d, *J* = 10.4 Hz, H-3), 5.20 (1H, d, *J* = 6.0 Hz, H-1''β), 4.95 (1H, d, *J* = 6.0 Hz, H-1''α), 4.19 (1H, m, H-2''), 4.16 (3H, s, OCH_3), 1.12 (3H, s, H-4''), 1.03 (3H, s, H-5'')

Compound **7**: δ 8.13 (1H, d, *J* = 10.0 Hz, H-4), 8.11 (1H, d, *J* = 2.4 Hz, H-1'), 7.66 (1H, s, H-5), 7.08 (1H, d, *J* = 2.4 Hz, H-2'), 6.42 (1H, d, *J* = 9.6 Hz, H-3), 4.56 (1H, dd, *J* = 10.0, 2.4 Hz, H-1''α), 4.34 (1H, dd, *J* = 10.0, 8.4 Hz, H-1''β), 3.78 (1H, td, *J* = 7.2, 2.4 Hz, H-2''), 3.15 (3H, s, OCH_3), 1.15 (3H, s, H-4''), 1.03 (3H, s, H-5'')

Compound **8**: δ 7.92 (1H, d, *J* = 9.2 Hz, H-4), 7.51 (1H, d, *J* = 8.4 Hz, H-5), 6.77 (1H, dd, *J* = 8.0, 2.4 Hz, H-6), 6.70 (1H, d, *J* = 1.6 Hz, H-8), 6.19 (1H, d, *J* = 9.6 Hz, H-3)

Compound **9**: δ 7.99 (1H, d, *J* = 9.6 Hz, H-4), 7.65 (1H, d, *J* = 8.8 Hz, H-5), 6.93 (1H, dd, *J* = 10.8, 2.4 Hz, H-6), 6.99 (1H, d, *J* = 2.4 Hz, H-8), 5.18 (1H, d, *J* = 7.6 Hz, H-1'), 3.68 (1H, d, *J* = 9.2 Hz, H-2'), 3.52 (1H, d, *J* = 9.2 Hz, H-3'), 3.39 (1H, d, *J* = 8.8 Hz, H-4'), 3.48 (1H, d, *J* = 9.2 Hz, H-5'), 3.80 (1H, d, *J* = 11.6 Hz, H-6'α), 3.58 (1H, d, *J* = 11.2 Hz, H-6'β), 5.11 (1H, d, *J* = 1.6 Hz, H-1''), 3.59 (1H, d, *J* = 1.6 Hz, H-2''), 3.49 (1H, d, *J* = 10.0 Hz, H-3''), 3.45 (1H, d, *J* = 9.6 Hz, H-4''), 3.75 (1H, d, *J* = 9.6 Hz, H-5''), 1.18 (3H, d, *J* = 6.4 Hz, H-6'')

S2. ^{13}C-NMR (100 MHz, DMSO-d_6) data of coumarins

	**1**	**2**	**3**	**4**	**5**	**6**	**7**	**8**
Position				δ_C				
1	-	-	-	-	-	-	-	-
2	159.7	159.9	159.8	159.9	159.6	159.6	160.3	161.3
3	114.2	114.3	114.2	114.2	112.6	112.5	114.4	111.4
4	145.3	143.2	142.6	145.3	139.8	139.7	145.7	144.5
5	113.9	114.2	113.8	113.8	144.3	145.3	114.1	129.7
6	125.9	125.8	125.8	125.9	114.4	114.4	126.2	113.4
7	147.9	147.9	147.8	147.8	149.4	149.6	148.0	160.4
8	131.9	126.9	130.8	131.5	127.2	126.9	131.7	102.5
9	142.4	139.2	142.6	141.9	143.1	143.2	142.9	155.5
10	116.4	116.9	116.4	116.4	106.8	106.9	116.7	111.3
1'	146.9	145.4	145.0	147.2	146.4	146.3	147.4	-
2'	107.1	107.1	107.1	107.1	105.7	105.6	107.4	-
5-OCH_3	-	-	-	-	60.8	60.8	-	-
8-OCH_3	61.0	-	-	-	61.3	-	-	-
1''	-	69.4	75.8	75.2	-	75.8	75.1	-
2''	-	119.7	72.9	76.2	-	76.7	76.5	-
3''	-	130.6	145.3	74.8	-	70.8	75.5	-
4''	-	17.8	111.9	19.8	-	24.4	22.3	-
5''	-	25.5	18.4	22.1	-	27.2	20.1	-
3''-OCH_3	-	-	-	-	-	-	49.2	-
